# Human Endogenous Retrovirus-K (HML-2)-Related Genetic Variation: Human Genome Diversity and Disease

**DOI:** 10.3390/genes14122150

**Published:** 2023-11-28

**Authors:** Wonseok Shin, Seyoung Mun, Kyudong Han

**Affiliations:** 1 NGS Clinical Laboratory, Division of Cancer Research, Dankook University Hospital, Cheonan 31116, Republic of Korea; wonseok@dkuh.co.kr; 2 Smart Animal Bio Institute, Dankook University, Cheonan 31116, Republic of Korea; 12200281@dankook.ac.kr; 3 College of Science & Technology, Dankook University, Cheonan 31116, Republic of Korea; 4 Center for Bio-Medical Engineering Core Facility, Dankook University, Cheonan 31116, Republic of Korea; 5 Department of Microbiology, College of Science & Technology, Dankook University, Cheonan 31116, Republic of Korea; 6 Department of Bioconvergence Engineering, Dankook University, Yongin 16890, Republic of Korea; 7 R&D Center, HuNBiome Co., Ltd., Seoul 08507, Republic of Korea

**Keywords:** human endogenous retrovirus-K, HERV-K, HML-2, genetic variation, genome diversity, diseases

## Abstract

Human endogenous retroviruses (HERVs) comprise a significant portion of the human genome, making up roughly 8%, a notable comparison to the 2–3% represented by coding sequences. Numerous studies have underscored the critical role and importance of HERVs, highlighting their diverse and extensive influence on the evolution of the human genome and establishing their complex correlation with various diseases. Among HERVs, the HERV-K (HML-2) subfamily has recently attracted significant attention, integrating into the human genome after the divergence between humans and chimpanzees. Its insertion in the human genome has received considerable attention due to its structural and functional characteristics and the time of insertion. Originating from ancient exogenous retroviruses, these elements succeeded in infecting germ cells, enabling vertical transmission and existing as proviruses within the genome. Remarkably, these sequences have retained the capacity to form complete viral sequences, exhibiting activity in transcription and translation. The HERV-K (HML-2) subfamily is the subject of active debate about its potential positive or negative effects on human genome evolution and various pathologies. This review summarizes the variation, regulation, and diseases in human genome evolution arising from the influence of HERV-K (HML-2).

## 1. Introduction

After decoding the human genome in 2001, we were confident that approximately half of the human genome is composed of transposable elements (TEs) and repetitive sequences [1]. These strongly influence polymorphisms and genomic variations in the human genome and have been implicated in human genome evolution and disease. Among them, human endogenous viruses (HERVs) and their associated genomic sequences occupy about 8% of the human genome, compared to the 2–3% of sequences that encode proteins [2,3].

During the evolution of the large primate genome, ancient exogenous retroviruses infected germline cells and accumulated in the primate genome as proviruses over a long period of time [3,4]. These successfully accumulated proviruses have similar sequence structures to exogenous viruses. Thus, they are known to be the result of exogenous retroviral infection of germline cells in the evolution of early primates [5,6,7]. They are broadly divided into class I, II, and III ERVs, each originating from a different retrovirus [5]. Each class has unique envelope proteins and different genetic characteristics, and exerts different effects on the host genome [8]. These classes are further divided into various families and groups [9]. Once converted to endogenous viruses, they tend to exhibit successful vertical transmission rather than horizontal transmission typical of infectious viruses [10,11]. Furthermore, they were considered a silent legacy in the evolution of the genome, having accumulated mutations in viral protein sequences and epigenetically altered and lost the ability to synthesize mature infectious retroviral particles [12,13,14].

We called them parts of junk DNA for a time and were unaware of their many functions [3]. In the 21st century, the field of molecular biology has expanded and made significant advances. Previous studies have shown that they exert a rich influence on us [7,15,16,17]. For example, they protect the host against infection by exogenous viruses and are involved in the physiological processes of many human cells [3,18,19]. In addition, they are significantly involved in the development and progression of many human diseases [3,7,17,20,21,22,23].

**Figure 1:**
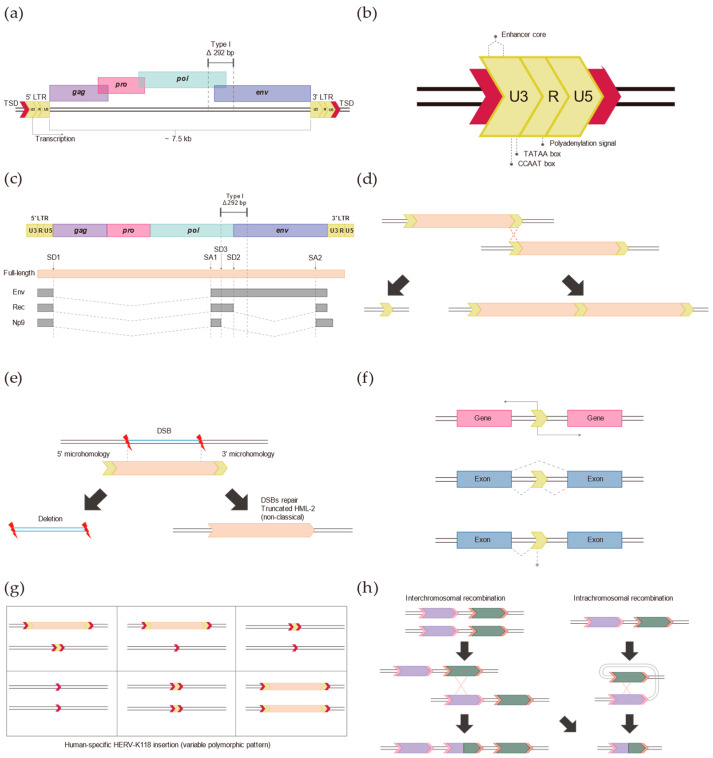
Typical structure and potential functions of HERV-K (HML-2) in the human genome. (**a**) General structure of HERV-K (HML-2). The typical full-length HERV-K (HML-2) is approximately 9.5 kb and is flanked by two long terminal repeats (LTRs). A typical HERV-K (HML-2) insertion forms a target site duplication (red box) by its insertion mechanism. The internal sequence has virus-like open reading frames (ORFs), largely composed of the *gag*, *pro*, *pol*, and *env* genes. This element can exist in two types (type I and II), with type I being a deletion of 292 bp at the border of the *pol* and *env* genes. (**b**) The LTRs exist as solitary LTRs through various genomic events within the human genome. Promoters, polyadenylation signals, enhancer cores, and putative factor-binding sites are present within LTRs and thus have the potential to play an important role in gene expression regulation and genomic variation. (**c**) HERV-K (HML-2) provides a splicing donor 3 (SD3) due to a 292 bp deletion at the border of the *pol* and *env* genes. As a result, type II expresses transcripts for the Env and Rec proteins and type I expresses transcripts that can produce the Np9 protein. (**d**) The LTRs after HERV-K (HML-2) insertion have very high sequence similarity; thus, homologous recombination can result in a duplicated form with two HERV-K internal regions and three LTRs, or it can exist as a solitary LTR. (**e**) Schematic of the non-classical insertion of HERV-K (HML-2) in the human genome. When double-strand breaks (DBSs) occur in ancient genomes, some existing sequences are deleted and truncated HML-2 sequences are inserted into the human genome without TSDs through microhomologies. (**f**) HERV-K (HML-2) drives changes in the human genome not only through its internal sequence, but also through its LTRs. LTRs have various regulatory elements within them and affect the human genome through antisense promoters, promoters, enhancers, polyadenylation signals, etc. They provide splicing donors and acceptors through their insertions. They also directly alter gene expressions through epigenetic changes. (**g**) HERV-K (HML-2) still show activity and due to their structural features, the specific insertion site exists in various forms within the diploid human genome. This means that they are still contributing to inter-individual genomic polymorphisms. (**h**) These elements are among the retrotransposons that were recently inserted into the human genome, so they have a very high degree of sequence similarity. These sequences can potentially cause genomic variation through interchromosomal recombination or intrachromosomal recombination.

Among human endogenous retroviruses, certain members of the HERV-K (HML-2) subfamily have joined the human genome most recently [24,25,26,27]. They are able to exist specifically in humans due to the timing of their entry, with low accumulation of mutations and epigenetic changes [28,29]. Thus, they are able to construct intact viral sequences and undergo active transcription and translation [3,30,31]. They are important mediators of early human embryonic development but are suppressed mainly in healthy adults [23,32]. Nevertheless, current research has shown that HERV-K is reactivated and implicated in various cancers, autoimmune, and other diseases [3,7,17,33,34,35,36,37]. In addition, their structural features and insertion mechanisms lead to polymorphisms in the human population genome [31,38,39]. To date, they still have a high potential for direct human genome alteration and regulation due to their specialized features [3,31,38,40]. Therefore, whether they are positive or negative in human genome evolution and pathology is a subject of rich discussion and research. This review summarizes the human genome changes, regulation, and diseases caused by the influence of HERV-K (HML-2) (Figure 1).

## 2. Typical HERV-K (HML-2)

Human endogenous retroviruses (HERVs) began to be discovered in 1981 [41]. They are exogenous virus-derived sequences that have stably settled into our genome [42]. ERVs have a structure similar to the proviruses of infectious viruses [43,44]. Therefore, they were considered to be the result of the exogenous retroviral infection of germ cells in the evolution of early primates [3,45]. They make up ~8% of the human genome and have accumulated about 203,000 proviruses over the generations [1,31]. They belong to retrotransposons, a type of TEs, and have typical mobility through a “copy and paste” mechanism [46,47,48]. Retrotransposons are so named because they are inserted into new genomic sites by transcription of an RNA intermediate [48,49]. They are also divided into LTR retrotransposons and non-LTR retrotransposons based on whether they contain long terminal repeat (LTR) structures [49,50].

HERVs belonging to the LTR retrotransposon subgroup are categorized based on the genome sequence of infectious retroviruses [6]. Typically, HERVs are divided into three distinct classes (I–III) [51]. These three different genera each originated independently (class I: gammaretrovirus-like and epsilonretrovirus-like, class II: betaretrovirus-like, and class III: spumaretrovirus-like) and include a variety of families [6,52] (Table 1).

However, the nomenclature for HERVs is somewhat controversial (i.e., they are not only present in humans). Furthermore, different names are used for other families and subfamilies, which leads to confusion. Nevertheless, the general nomenclature of the well-known HERVs utilizes the tRNA molecules used for reverse transcription priming of retroviruses. Among them, HERV-K has a private binding site (PBS) region complementary to the K-amino acid (lysine)-tRNA molecule [53]. They were first reported in 1986, and this class II HERV-K supergroup is now known to consist of 11 subgroups [43]. These subgroups are labeled HML-1 through HML-11. Here, HML (human MMTV-like) is closely related to MMTV (mouse mammary tumor virus), which has been implicated in the vertical transmission of mammary cancer in mice [3,43,54].

Among them, the HML-2 subgroup is the most recent to join the human genome since the human-chimpanzee divergence approximately 6 million years ago. It has been reported to act as an active TE [43,55]. As such, they retain a relatively high degree of sequence conservation, which strongly predicts their transcriptional activity and has long been a subject of observation [35,56]. These HML-2s were further categorized into three subgroups based on phylogenetic analysis using LTR sequences: LTR5A, LTR5B, and LTR5Hs, of which LTR5Hs were reported to be the youngest [57]. Thus, LTR5Hs are the only group with human-specific insertions [58]. As is the case with almost all retrotransposons, most HML-2s have lost their ability to replicate on their own due to the continuous accumulation of mutations and deletion and recombination of their internal sequences since they joined the host’s genome [59]. Despite their inability to generate infectious viruses, they have been reported to remain transcriptionally active and have maintained the production of retrovirus-like particles by encoding functional retroviral proteins, suggesting that they still have the potential to be active retrotransposons [3,60].

These HERVs have a similar structure to the proviral forms of infectious viruses. A full-length HERV element is approximately 9.5 kb and contains four core viral genes (*gag*, *pro*, *pol*, and *env*) in its internal region, flanked by two long terminal repeats (LTRs) [17,31]. The core structural gene of the internal region of the HERV element, *gag*, encodes group-specific antigens such as capsid and nucleocapsid proteins [3]. The core gene *pro* encodes a deoxyuridine 5′-triphosphate nucleotide hydrolase (dUTPase) and a protease [32]. The core gene *pol* consists of a reverse transcriptase domain [3]. It is a viral enzyme that synthesizes viral RNA into viral cDNA from a template [61]. The core gene *env* is an envelope domain that encodes an envelope glycoprotein that is important for membrane fusion and receptor recognition [61]. The *env* gene also involves another HERV family of LTR retrotransposons [3,51,62]. The HML-2 subgroup of the HERV-K supergroup is divided into type I and type II based on the presence or absence of a 292 bp sequence at the *pol*-*env* gene boundary in the internal region [7,17]. Only type II contains the 292 bp sequence. Therefore, the potential to have an intact proviral gene lies with type II. The type II HML-2 provirus encodes *env* intact and can also express accessory Rec proteins that bind to their transcripts and facilitate nucleocytoplasmic transport [3,24]. Type I HML-2 viruses lose the splice donor site due to a deletion of 292 bp at the *pol*-*env* boundary and are unable to encode Rec or Env [3,40,63]. However, another splice donor site located upstream of the 292 bp deletion region can be utilized to express the Np9 protein [3]. This protein is known to have no physiological function in the replication of HML-2 [29].

## 3. Long Terminal Repeats (LTRs)

The pair of LTRs on the flanking side of the HERV’s internal body contain many regulatory elements such as promoters, enhancers, and polyadenylation signals that play a role in retroviral gene expression [64]. In addition, they have the potential to significantly influence host gene expression [3,31]. They are present in much higher numbers than typical HERV insertions [3,65]. Therefore, their regulatory function has been of interest for some time due to the nature of their structure [66,67]. In fact, most LTRs have their regulatory functions epigenetically suppressed [3,67]. However, these retroviral LTRs have been estimated to initiate 10 times more transcription than typical promoters, even in healthy normal cells [3,32].

The structure of the full-length HML-2 provirus has two LTRs flanking it [17]. This LTR consists of a 3′ unique element (U3), a repeat element (R), and a 5′ unique element (U5) in the 5′ to 3′ direction [68]. The U3 region is primarily responsible for promoter and enhancer functions and contains binding sites for various transcriptional regulators that control the expression of retroviral genes [68]. This region serves as the beginning of gene transcription and provides a region where specific transcription factors bind to various sequence elements that regulate gene expression [69]. The R region plays an important regulatory role within the LTR and contains elements related to RNA stability and transcription termination [70]. This region is associated with the transcriptional start of the gene and helps in the production of the primary transcriptional replicas [70]. The U5 region forms the 5′-end of the transcript, marks the end of the transcription event, and plays an important role in post-transcriptional replication [71]. They ensure the stability of the gene and play an essential role in the process of gene duplication and insertion [3,7]. Thus, they act as important regulators of gene expression due to their structural characteristics and influence host gene expression [72].

Among them, LTR5Hs are one of the human-specific TEs that have been reported to be involved in regulating the expression of our genes due to their ability [58]. In particular, previous studies have reported that these LTRs play a positive role in the body by inducing the expression of silenced tumor suppressor genes [7,14,73]. Previous studies have demonstrated that the HML-2 LTR sequence can induce antisense transcription under selected conditions [28,74,75]. The ability of HML-2 LTR to insert in both forward and reverse orientations has been experimentally validated through luciferase assays, suggesting the potential of bidirectional promoter activity in the absence of a reporter gene’s promoter [75]. Bioinformatics analysis has reported the functionality of the antisense promoter in the LTR, indicating its influence on gene expression across diverse regions of the human genome [74,75]. Consequently, this functionality can modulate cellular gene expression in multiple ways, impacting the expression patterns of the human genome and potentially conferring selective advantages during genomic evolutionary processes [76]. Additionally, the insertion of HML-2 LTR can introduce new genes or gene regulatory elements into the human genome, fostering diversity and, in some instances, leading to the acquisition of novel functionalities [77]. Conversely, this can be associated with detrimental effects, contributing to various human diseases [3]. In conclusion, the presence of an antisense promoter in HML-2 LTR implies a significant correlation with the evolution of the human genome [31,77].

In fact, relatively more studies show that LTR adversely affects humans [3,17]. The hypomethylated state of HML-2 LTR may be linked to increased activation in specific cancer cells [7,78]. It has been extensively reported to be associated with the activation of various cancers such as colorectal cancer, breast cancer, glioblastoma, pancreatic cancer, and prostate cancer [3,79,80,81,82]. Such activation can enhance the survival and mobility of cancer cells, potentially impacting patients’ treatment response and survival rates [83]. The hypomethylation of HML-2 LTR induces the activation of the corresponding promoter, thereby affecting the expression of adjacent genes. In relation to the transformation of breast epithelial cells, it has been reported that the activation of the 5′ LTR promoter of HERV-K (HML-2) tends to be specifically found in cancer cells [7,23]. These findings indicate that interactions with specific signals within or outside certain cells can play a crucial role in activating the LTR promoter [84]. Additionally, as the LTR region provides various transcription factor binding sites, mutations in this sequence can correlate with gene expression [85]. Furthermore, a 2016 study by Manghera et al. confirmed the presence of a functional interferon-stimulated response element (ISRE) in the promoter region of HERV-K [86]. This element suggests that the expression of HERV-K can be regulated by inflammatory cytokines, particularly interferons [86]. Interferons are proteins produced in response to abnormal conditions such as viral infections and activate the defense mechanisms of cells [86]. The existence of ISRE in the promoter region of HERV-K implies that the presence of cytokines-like interferons can increase the expression of HERV-K [3]. This suggests a potential impact on the onset and progression of autoimmune reactions and autoimmune disorders. The role of LTR as a potent regulatory element contributing to various diseases and related genetic alterations is becoming increasingly evident.

## 4. Insertion of HERV-K (HML-2) in the Human Genome

To date, various human reference genomes have been publicly released. Although the latest human reference genome, hg38, is available, hg19 continues to be widely used [87]. This predominance is mainly due to the extensive base of existing research and datasets grounded on hg19, facilitating the maintenance of data consistency. However, a notable limitation of this reference genome is that it predominantly reflects the genetic variations of European Caucasians, thereby not comprehensively covering the diversity present in other ethnic groups [88]. Despite this limitation, it serves as an excellent consensus for analyzing and researching the genome.

Various studies have been conducted to determine the accurate number of HERV-K (HML-2) insertion sites and, due to the structural characteristics and polymorphism of HERV-K (HML-2), the results may vary. Several previous studies have reported that at least 1000 HERV-K (HML-2) elements are inserted in the modern human genome [3,89]. Most of these elements have lost their internal sequences through homologous recombination between LTRs, resulting in solitary LTRs [31]. According to a 2011 study by Subramanian et al., solitary LTRs are approximately ten times more common than proviral integrations with full-length or truncated internal sequences [43]. These relatively recent insertions after the human-chimpanzee divergence contribute to genomic variation between humans and chimpanzees through human-specific insertions and genomic rearrangements. In addition, various previous studies have measured HERV-K (HML-2) insertion polymorphisms in the human population and demonstrated that these elements could generate genomic variation within the human species [26,31,90]. This suggests that retrotransposition has occurred in the human genome since the human-chimpanzee divergence resulting in polymorphisms between human populations and between individuals. Analysis of inter-species sequence homology by comparative genomics identified polymorphism patterns of HERV-K (HML-2) and, in particular, confirmed that approximately half of the human-specific HERV-K (HML-2) is not fixed in the human population, suggesting that HERV-K (HML-2) contributes to individual genomic diversity in the human population [31].

The human-specific HML-2, a subgroup of HERV-K, is unique in that it integrated into the genome after the divergence of humans and chimpanzees [3]. Consequently, it induces genomic differences between humans and chimpanzees through species-specific insertions and genomic rearrangements [31]. Pinpointing the exact number of human-specific insertions is challenging due to variances in the definition and detection criteria of insertion sites across different studies and the continual discovery of new insertion sites. This variability arises from differences in approaches, methods, and individual variability and the fact that these elements still retain the capability for retrotransposition [45,91,92]. Nevertheless, various studies have reported over 30 human-specific HERV-K insertions to date [3]. These exhibit more polymorphism than other human-specific retrotransposons [31,93].

The HML-2 elements, integrated most recently into the human genome after the divergence from chimpanzees, exhibit genetic polymorphism within human populations, potentially influencing various biological characteristics such as disease susceptibility and immunity [3]. This implies their capacity to engender genetic variations both within the human species and between different human populations [31]. Furthermore, the increasing phenomena of human-specific HML-2 insertions, not found in our closest primates, suggest their contribution to human genomic evolution by mediating gene expression regulation and chromosomal rearrangements [43]. The HML-2 elements are present across all genetic loci in the human genome and can exhibit variations in copy number, distribution, and structure among individuals [17]. These interindividual variations can arise due to various influences, including insertion polymorphisms, duplications, deletions, solitary LTRs, and single nucleotide polymorphisms (SNPs) [26,92].

HERV-K (HML-2) exhibits insertion polymorphism among individual humans. Moreover, due to their structural characteristics, they can manifest more abundantly than other retrotransposons in terms of presence, absence, solitary LTR, and duplication [31]. This can lead to structural and functional changes in genes and regulatory regions, affecting gene expression [48,93]. Consequently, such insertion polymorphism can affect phenotypic diversity and potentially influence the susceptibility of an individual to specific diseases [3]. In addition, SNP variants in HERV-K (HML-2) can affect the activity and function of host genes [94]. In particular, depending on where the SNP occurs, it can affect the structure and function of proteins in various ways, including altering the binding sites of transcription factors and microRNAs [95].

Loci such as HERV-K109, HERV-K118, and HERV-K134 exhibit human-specific insertions and have directly contributed to human genome evolution [3,31]. Notably, these regions display insertion polymorphism in individual human genomes, existing in three forms: presence, pre-insertion state, and solitary LTR [40,96]. Additionally, these regions are present in the human genome either in heterozygous or homozygous states, indicating that they are not yet fixed [3,31]. Currently, these HERV-K (HML-2) loci are being reported to have associations in various cancer and disease studies [3,62,94,97,98,99]. The diverse forms of variations, namely SNP, duplication, deletion, and insertion polymorphism, influence the activity, expression, and functional alterations of HERV-K (HML-2), ultimately impacting the host physiologically [3].

HERV-K113, HERV-K115, and HERV-K119 are known to be capable of producing intact viral genomic structures [31,100]. This indicates the potential of these HERV-K (HML-2) elements to encode functional proteins necessary for their new insertions. In fact, previous studies have confirmed that HERV-K113 and 115 possess full length in the human genome and induce polymorphism [96]. Polymorphism of HERV-K119 was also confirmed to occur in human populations, but it exists in a different form from other elements. Specifically, the polymorphism of HERV-K113 and 115 is determined by the presence or absence of HERV-K. In contrast, in the case of HERV-K119, solitary LTR forms exist due to interchromosomal recombination between the 5′ and 3′ ends of both LTRs due to a mismatch of sister or non-sister chromatids. Based on these results, it is hypothesized that the HERV-K119 element was inserted relatively long ago compared to the other two elements (HERV-K113 and 115) [31]. These intact and full-length HERV-K elements possess sufficient potential to cause human diseases [101]. Indeed, several studies have reported the presence of transcribed and encoded proteins by HERV or HERV-K in tumor and autoimmune disease patients, suggesting a potential role in human diseases [3,42,78].

The typical HERV-K (HML-2) insertion mechanism is mediated by the virus’s integrase [17]. This enzyme cleaves the host DNA and inserts the viral cDNA. During this process, the enzyme generates sticky ends on both sides of the DNA, and the cleaved DNA forms a target site duplication (TSD) as it is replicated by the host cell’s DNA repair mechanism. Its presence is strong evidence of a typical HERV-K (HML-2) insertion. The TSD is 5–6 bp, except in special cases [26,102].

## 5. Discovery of Non-Reference HERV-K (HML-2)

Full-length forms of HERV-K (HML-2) are known to still potentially have retrotransposition function [17]. Most HERV-K (HML-2) elements do not generate factors that cause horizontal propagation and are inactivated by mutations that have accumulated over time [103]. However, in 2015, Contreras-Galindo et al. reported that modern HERV-K (HML-2) viruses can be transmitted to other cells via reverse transcription [30]. Furthermore, in 2013, Hohn et al. discussed that the existence of infectious HERV-K (HML-2) proviruses cannot be completely ruled out, although their prevalence is low [32]. Certain full-length HERV-K (HML-2) proviruses, such as HERV-K113, HERV-K115, and HERV-119, have intact viral sequences within the provirus, allowing full expression of the viral genes [3,31]. Based on these features and previous reports, the potential for retrotransposition to novel regions is substantial. Therefore, effective and economical methods to localize human HERV-K (HML-2) are needed to reveal novel insertion regions, insertion polymorphisms, and causal relationships between human diseases that have not been reported in new reference genomes.

Recent advances in applying next-generation sequencing (NGS) technologies, including whole genome sequencing, transcriptome sequencing, exome sequencing, and microRNA profiling, have contributed significantly to genomic research. However, the identification of non-reference retrotransposons in specific regions is limited by rearrangements [88]. For example, when mapping the human reference genome, resequencing additional reads may be discarded by bioinformatics algorithms. Due to the repetitive sequence nature of the transposable elements (TEs) in short reads, they may map to similar regions rather than their original position [104]. Another limitation is that repetitive sequences, such as poly-A tails, reduce the quality of the sequencing reads, making effective data utilization difficult [88]. Thus, despite several methods such as ME-can, ATLAS, and SIMPLE being reported to identify non-reference retrotransposon insertions in human individuals, it has been challenging to detect newly inserted mobile elements and their polymorphisms using NGS methods [54,88,105,106,107]. In fact, de novo assembly using long-read sequencing is a suitable method for effective identification, but it is very costly and time-consuming [104]. Therefore, it is important to develop an effective and efficient TE discovery method to identify novel insertions and their roles in the human genome.

## 6. Role of Non-Classical HERV-K (HML-2) Insertions in the Human Genome

Very rarely in the human genome, HERV-K (HML-2) exists as an atypical insertion, similar to other retrotransposons [83]. These atypical HERV-K (HML-2) insertions are characterized by the absence of both the 5′ and 3′ end regions and do not contain a TSD, which is evidence of a typical insertion [31]. They are associated with specifically targeted site deletions in human genome evolution [48,93]. Their deletion sites can be identified in the human genome by comparing the human-specific HERV-K (HML-2) insertion flanking with the corresponding pre-insertion sequence in primate genomes [31]. The existing primate sequence of these atypical insertions in the human genome has been deleted, varying from as little as 6 bp to about 10 kb [31]. Their role has been reported to be associated with double-strand break (DSB) repair mechanisms to maintain the stability of the human genome [31]. Meanwhile, DSB repair mechanisms to maintain an intact genome in eukaryotic cells can repair non-allelic homologous recombination (NAHR) or non-homologous end-joining (NHEJ) [108]. The evidence of these insertions is the presence of microhomologies [31]. This is because when NHEJs occur, microhomology is required for DSB repair with HERV-K (HML-2) [109]. The presence of 1 to 7 bp of homology sequences in the HERV-K (HML-2) sequence on both the 5′ and 3′ ends and in existing primate genomes may play a repairing role [31]. This event appears to play a similar role to other existing retrotransposons in the evolution of the human genome [48,110]. In conclusion, it is proposed that they act as wound-healing bands in the human genome.

## 7. Proteins and Particles of HERV-K (HML-2)

HERV-K (HML-2) encodes several proteins of its own [17]. Some affect the physiological function of the host cell. The HERV-K (HML-2) Env protein is an outer membrane glycoprotein that is thought to have multiple pathogenetic roles that may affect the function of the immune system [3]. It may be involved in cell–cell interactions, cell proliferation and survival, and intercellular signaling [78]. In particular, there are various reports that this protein may be involved in human disease states such as autoimmunity and cancer [29,62,97,111,112]. Indeed, it has been shown to induce cytotoxic and apoptotic responses in innate and adaptive immunity and to exhibit properties inhibiting immune activation [3,8,29]. In addition, a 2013 study by Huang et al. reported that Env proteins may contribute to tumorigenesis by promoting cell–cell fusion in melanoma [113]. In 2016, Zhou et al. reported that when this protein is artificially regulated in breast cancer cells, it is involved in oncogene expression, cell proliferation, migration, and invasion [33]. The HERV-K Env protein is significantly more expressed in most tumors than surrounding normal tissues. Similarly, in ovarian cancer, the HERV-K Env protein and its associated cell surface proteins may serve as novel tumor targets for diagnosis and treatment [114]. Meanwhile, within the complex human immune system, HERV-K proteins have the potential to fine-tune the immune response to tumors by providing antigenic epitopes that can be recognized by T and B cells [115]. They have been implicated in antibody production in diseases such as certain cancers, immune disorders, and brain disorders, and their presence can be linked to disease severity [3,83,102,116].

HERV-K (HML-2), as it is widely known, is divided into two types. It is divided into type I and type II based on the presence or absence of a deletion of 292 bp of the *pol*-*env* boundary. Type II has the 292 bp intact, so it can still encode the *env* gene and express the Rec protein [3,7]. Type I, on the other hand, cannot express Env and Rec proteins but can express the Np9 protein [117]. Although Np9 and Rec proteins are derived from HML-2 in different conformations, they can interact with various intracellular proteins to affect cell function and physiology [3]. Their accessory Rec proteins bind to the negative regulator hSGT to increase the activation of the androgen receptor (AR) [118]. This enhances the transcription of AR-dependent genes as well as the expression of HERV-K (HML-2), which is involved in a vicious cycle leading to cancer progression. Both Rec and Np9 appear to be present in a variety of normal human tissues. Still, some studies have suggested that the promyelocytic leukemia zinc finger (PLZF) protein, a transcriptional repressor of the c-myc (proto-oncogene gene), is involved in the development of cancer by repressing c-myc and interacting with Rec and Np9 [27]. In 2019, Rigogliuso et al. reported that expression of the HERV-K (HML-2) protease (*pro*) affects a variety of proteins, which in turn affect cellular function and are implicated in disease [119]. This network of HERV-K (HML-2) proteins has been shown to affect human diseases and various cellular pathways. However, a lot of research and different perspectives are still needed.

HERV-K (HML-2) is generally not reported to be infectious. However, its features are sufficient to produce virus-like particles. These HERV-K (HML-2)-like particles have been reported in various diseases, and their expression is usually increased [3,120,121]. They were discovered in cancer-derived cell lines and identified in breast, ovarian, and melanoma cells [30,122]. Human immunodeficiency virus (HIV)-1 infection promotes the activation of HERV-K (HML-2) to form viral particles [123]. It is believed that T-cell responses to the HERV-K (HML-2) protein help suppress HIV-1 viral load [3]. This may have positive implications for the development of therapies against HIV-1. Virus-like particles have also been found in the brain tissue of patients with neurodegenerative diseases such as Lou Gehrig’s disease (amyotrophic lateral sclerosis; ALS) [99]. Several studies have also reported increased expression of HERV-K (HML-2) and particle formation in the brains of patients with autism [3,61]. These various studies broaden our view of the relationship between HERV-K (HML-2) expression and related proteins and provide robust evidence for developing treatment, diagnosis, and prevention strategies for various human diseases. Studying HERV-K (HML-2) protein and its associated factors can provide good insights into the understanding of human diseases and is expected to play an important role in developing strategies to overcome human diseases.

## 8. Association between HERV-K (HML-2) and Human Diseases

HERV-K (HML-2) has been directly involved in human genome evolution and diversity since its integration into the human genome [77]. It has been co-existing with the human genome for a long time, can express its transcripts and proteins, and has been found in various cells, including stem cells, immune cells, etc. [78]. Its diverse structural and functional capabilities have led to various relationships with host cells [32]. It has played important roles in normal physiology. HERV-K (HML-2) influences cellular function and biological pathways by promoting or repressing the expression of specific genes through its promoter and enhancer functions, which can regulate gene expression [124]. In addition, they are crucial in normal pregnancy, playing a positive role during placental development [3,125]. Some HERV-Ks (HML-2s) are expressed in placental tissue, where they are involved in the cell fusion process necessary for forming and maintaining the placenta [3]. They also participate in the immune response through antivirals. This affects the development and function of the immune system, regulating how the body responds to pathogens [3]. Some studies have suggested that HERV-K (HML-2) may affect nervous system development, which has been linked to its expression in certain regions of the brain [3]. It has been integrated into the host genome during evolution and contributed to increasing the diversity and complexity of the genome. It may have been integrated into the host’s biological functions as part of an evolutionary adaptation.

**Figure 2:**
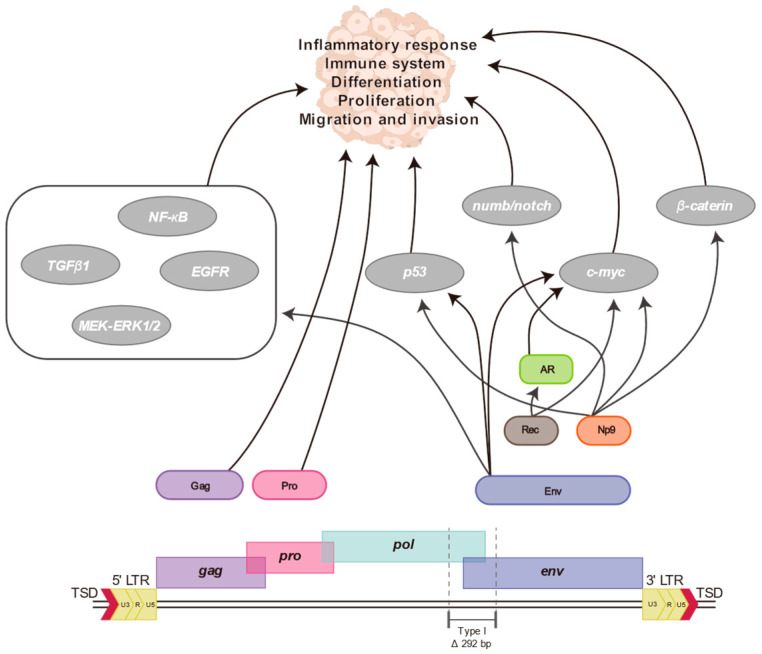
Diagram of HERV-K (HML-2) and its relationship to disease. The proteins expressed by this element, Gag, Pro, Env, Rec, and Np9, interact with various host genes to induce abnormal cellular physiological responses. The black arrows indicate their respective associations. These proteins are known to affect human diseases, including various malignancies, by influencing inflammatory responses, the immune system, and cell growth and progression.

However, its direct involvement in human disease has recently been recognized, leading to a great deal of follow-up research. Various studies have shown that the viral protein of HERV-K (HML-2) is involved in the progression of human diseases (Figure 2). Its Env protein is involved in epithelial mesenchymal transition (EMT) and intercellular fusion, and its accessory proteins, Np9 and Rec, have been suggested to be involved in tumorigenesis through regulation of the c-myc gene and androgen receptor [3]. These proteins regulate signaling pathways involved in cell growth and proliferation. Its LTR regions contain various regulatory elements such as bidirectional promoters, enhancers, splicing donors/acceptors, and poly A signals, which can significantly influence proximal genes [3]. In addition, the presence of interferon-stimulated response elements (ISREs) in the region suggests that their expression is regulated by inflammatory cytokines [3,86]. LTRs are repetitive sequences and chromosomal rearrangements through them contribute to aberrant gene expression profiles [126]. HERV-K (HML-2) has been implicated in human disease due to its structure and properties.

It is important that they are involved in cell signaling during tumorigenesis and that these proteins cause autoimmune errors in the host cell [29,116]. In a previous study, it was shown that knocking out the expression of the Env protein of HERV-K (HML-2) with shRNA blocked the proliferation, migration, and invasion of breast cancer cells. It was reported that shRNAenv transduction attenuated the tumorigenic ability of breast cancer cells and prevented metastasis. It wasnsuggested that key upstream regulators p53, TGF-β1, and MYC were affected [33]. In this study, the HERV-K *env* expression vector was used to overexpress the HERV-K (HML-2) *env* gene in shRNAenv-transducted breast cancer cells and it was found that the MEK-ERK signaling pathway was restored. Moreover, CDK5, which phosphorylates p53, was upregulated in cancers and p53 was downregulated when HERV-K was overexpressed. This suggests that the HERV-K (HML-2) Env protein plays an important role in the tumorization of breast cancer cells [33]. On the other hand, HERV-K (HML-2) has a structurally diverse transcriptional regulator and is likely to be involved in human genome variations such as ectopic recombination, DSB repair, insertion-mediated deletions, and gene conversion due to its special characteristics [17]. These factors make normal gene regulation in the host cell impossible and cause genetic instability [3]. Thus, HERV-K (HML-2) and other retrotransposons are potential pathogenic factors in human disease [127]. While much research is still needed to unravel the complexity of disease etiologies and their interrelationships, this is important information for understanding and overcoming human disease.

The polymorphisms and SNPs of HERV-K (HML-2) in human cancers are associated with an increased risk of tumor development. HERV-K (HML-2) expression has been observed in several types of cancers, including breast, prostate, and ovarian, and has been reported to be associated with human tumors [43,82]. In particular, it has been reported to be involved in cancer gene activation, abnormal cell proliferation, and immune regulation in tumorigenesis [5]. In addition, its transcripts and proteins have been reported to be upregulated in various solid and liquid tumors [83,128]. For example, a previous study identified HERV-K (HML-2) transcripts in breast cancer cell lines and breast tumor tissue, which were not found in benign breast tissue. This selective expression suggests that HERV-K (HML-2) has the potential as a biomarker of malignancy [129]. Cellular expression of the HERV-K (HML-2) Env protein was observed in ovarian cancer cell lines and tissues. The presence of this protein on the cell surface suggests that cancer cells may be utilizing cell–cell interactions and cell signaling pathways. In addition, antibodies to HERV-K (HML-2) were detected in samples from ovarian cancer patients [114]. For germ-cell tumors, anti-HERV-K (HML-2) antibodies were found in 67% of patients, suggesting that these serologic antibody levels could be used as a possible marker of disease progression [130]. The HERV-K (HML-2) Gag protein has been reported to be increased in Seminoma. The increase of this protein in Tera 1 cells and the high antibody retention in patients suggest that HERV-K (HML-2) is active in cancer [131]. An average 61.56% decrease in HERV-K (HML-2) methylation was observed in colon cancer patient samples, and Env protein was expressed in tumor tissue but not in surrounding normal cells [132]. It has been reported that the HERV-K (HML-2) *env* transcript was detected in several pancreatic cancer cell lines [133]. In addition, previous studies have shown that the transcript, Env, and Gag proteins of HERV-K (HML-2) are expressed at high levels in the plasma of lymphoma patients and that transcription of the HERV-K (HML-2) *gag* gene is ten times higher than usual in leukemia blood samples [98,134]. Increased expression of HERV-K (HML-2) and its constituent proteins has been observed in hepatocellular carcinoma (HCC) and glioblastoma (GBM), and in HCC they have been found to be negative prognostic indicators [82,135]. Increased HERV-K (HML-2) expression in these various cancers eventually leads to cell proliferation, differentiation, immune response, tumorigenesis, and inflammatory response using the host cell signaling system and molecular interaction.

The genetic variants of HERV-K (HML-2) and their variations in the human genome contribute to population differentiation and human genomic diversity [3]. Therefore, they can cause interindividual differences in disease prevalence, distribution, and susceptibility [3]. These inter-individual differences can potentially manifest as phenotypic differences by affecting gene expression and regulation in host cells [3]. Eventually, this inter-individual variation can lead to differences in infection and immune susceptibility in a population, resulting in a more complex causal relationship with disease. Various genetic variants cause direct changes in HERV-K (HML-2) expression and host cell status. This significantly impacts cancer susceptibility by altering the immune level of host cells and changing the activity of tumor suppressor genes and oncogenes.

HERV-K (HML-2) has been implicated in autoimmune and inflammatory diseases [62,86,116,136]. Previous studies have reported that HERV-K (HML-2) transcripts and proteins activate the immune response and eventually produce inflammatory molecules [29,37,69]. This suggests that they may affect immune function and increase tissue damage. This abnormal immune response can lead to allergies and immune diseases. HERV-K (HML-2) expression can trigger innate and adaptive immunity [29]. Associations between autoimmune diseases such as multiple sclerosis (MS) and rheumatoid arthritis (RA) have been reported [32,137]. MS is a chronic autoimmune disease of the central nervous system and is characterized by the destruction of myelin [138]. Increased HERV-K (HML) expression has been observed in the brain and spine of MS patients and is associated with inflammation and damage to brain tissue [3]. In addition, RA leads to inflammation and destruction of the joints. Increased HERV-K (HML-2) expression has been observed in RA patients [124]. This suggests that the human immune system recognizes the protein of HERV-K (HML-2) and promotes an autoimmune response [139]. Amyotrophic lateral sclerosis (ALS) is a neurological disease characterized by the pathological degeneration of motor neurons in the central and peripheral nervous system [140]. Various studies have suggested an association between HERV-K (HML-2) and ALS [141,142,143]. Increased transcript and protein expression of HERV-K (HML-2) has been reported in brain and spinal cord tissue from ALS patients [142]. In particular, the presence and activation of HERV-K in motor neurons have been identified [95]. In ALS, the Env protein of HERV-K (HML-2) may act as a neurotoxin and negatively affect the degeneration of motor neurons [99]. In some previous studies, HERV-K (HML-2) overexpression was found in the brain tissue or blood of patients with schizophrenia [3]. This suggested that HML-2 affects neurodevelopment, synaptic plasticity, immune response, and inflammation, potentially linking it to schizophrenia [3].

The functional relevance of the pathological responses associated with HERV-K (HML-2) still needs to be fully understood. Nevertheless, studies continue to report increased pathological responses to HERV-K (HML-2) in various human diseases. This includes the aberrant expression of the HERV-K (HML-2) protein, production of antibodies against the protein, and viral particle production, as well as various physiological associations with host cells and their consequences. Understanding the interplay between HERV-K (HML-2) pathological responses and human disease has now entered a critical stage in various research fields. Therefore, researchers are studying HERV-K (HML-2) component genes and their complex and extensive interrelationships with human genes to gain insights into disease. In addition, there is an emphasis on understanding how HERV-K (HML-2) affects the onset and progression of disease by studying the mechanisms underlying the pathological response. Furthermore, studying how the aberrant expression of the HERV-K (HML-2) protein affects the human immune system may be of major interest. In particular, a deeper understanding of the formation of antibodies against this protein and the resulting response is needed. Further studies will provide a more precise experience of the impact of HERV-K (HML-2)-associated pathological responses on the pathogenesis and development of human disease, which is expected to play a crucial role in developing disease management and treatment strategies.

## 9. HERV-K (HML-2) Transcriptome and Human Diseases

The aberrant viral transcript expression of HERV-K (HML-2) is an important factor associated with several human diseases. This transcript expression is predicted to contribute significantly to the diagnosis or prognosis of certain diseases. In a recent study, HERV-K (HML-2) expression profiling identified the higher expression of HERV-K (HML-2) compared to normal surrounding tissues in almost all types of tumors [144]. Specifically, the HERV-K (HML-2) *env* gene was highly expressed in breast, melanoma, kidney, prostate, cervical, esophageal, and colorectal cancers. However, in osteosarcoma, it did not show significant differences from normal tissue. In liver cancer cells, the expression of HERV-K Env protein was upregulated in older individuals. However, it was suggested that additional data is needed to confirm significant differences by age and gender [145].

In cancer, for example, the reverse transcriptase of HERV-K (HML-2) can be expressed in early malignant breast cancer and is a candidate for a novel prognostic marker for breast cancer [146]. Other previous studies have shown that HERV-K (HML-2) *env* transcript expression in breast cancer tissues is significantly higher than in normal tissues, suggesting its potential as a diagnostic marker for breast cancer [147]. High HERV-K (HML-2) Env protein expression is also associated with breast cancer progression and negative outcomes. This suggests that the HERV-K (HML-2) *env* region could be used as a diagnostic marker for breast cancer. Furthermore, increased expression of HERV-K108, HERV-K109, HERV-K113, and HERV-K115 was observed in a subset of basal breast cancer and was associated with a higher frequency of recurrence and metastasis [148]. These loci suggested that they may be important targets for developing cancer vaccines or immunotherapy. Aberrant expression of HERV-K (HML-2) has been consistently observed in various cancer types, not only breast cancer. HERV-K (HML-2) antibodies were increased in the blood of patients with early stage breast cancer, and antibody levels were further increased in patients at risk of metastasis [149]. These antibodies, like transcripts, showed promise as cancer markers, suggesting that antibody responses to the HERV-K protein may provide important prognostic information for breast cancer patients [150]. The transcription level of the HERV-K (HML-2) *env* gene was found to be significantly increased in the blood of patients with various types of lung cancer compared to healthy controls [151].

In a previous study, it was found that the transcription level of HERV-K (HML-2) *env* was significantly higher in adenocarcinoma than in squamous cell carcinoma (SCC) and small cell lung cancer (SCLC) [151]. These results were obtained using blood, suggesting that it could be utilized as a non-invasive blood-based lung cancer marker. In hepatocellular carcinoma (HCC), a significant increase in the expression level of HERV-K (HML-2) was observed compared to normal tissues [82]. It was associated with cirrhosis, tumor differentiation, and TNM stage; higher expression levels were associated with poorer survival. Therefore, it was suggested that the expression level of HERV-K (HML-2) in HCC could be used as a prognostic factor. In prostate cancer, various studies have been reported. In men with prostate cancer, the expression of HERV-K *gag* was significantly higher in malignant lesions compared to benign lesions or normal tissue [34,35,152]. Traditionally, prostate-specific antigen (PSA) testing has been used as an important tool to diagnose prostate cancer, but it has limitations due to its low specificity and high false-positive rate [153]. Therefore, combining PSA testing with non-invasive testing of specific HERV-K (HML-2) expression levels may be more effective in diagnosing prostate cancer. In melanoma, various studies have suggested that increased expression of the protein and transcript encoding HERV-K (HML-2) may contribute to melanoma development [36,154]. HERV-K (HML-2) at specific locations reported to be associated with melanoma may transcribe their *gag* and/or *env* genes [155]. Furthermore, their activation in melanoma cells has been shown to be associated with progression to more malignant tumor forms. Overexpression of HERV-K (HML-2) has been reported to be associated with various hematologic malignancies [3,7]. In pediatric acute myeloid leukemia (AML), high HERV-K *env* gene expression levels were detected, suggesting that it contributes to disease development [37,156]. In addition, increased levels of Np9 expression were found in chronic lymphocytic leukemia (CLL) and significant levels of HERV-K expression were found in bone marrow samples from acute lymphoblastic leukemia (ALL) patients [37,157]. These results support that HERV-K expression is associated with leukemogenesis. In addition, increased expression of HERV-K (HML-2) has been reported in malignant lymphomas, soft tissue sarcomas (STS), and ovarian epithelial tumors [3,78,158]. The expression of HERV-K (HML-2) transcripts in various cancer types has been the subject of ongoing research interest. This suggests that there is an important relationship between them. Therefore, more detailed and in-depth studies are needed to diagnose and overcome various human cancers.

The aberrant expression of the HERV-K (HML-2) gene itself has been found in many other diseases besides cancer. Previous studies found that the *gag*, *pol*, and *env* transcripts of HERV-K (HML-2) were increased in the brain tissue of ALS patients [3,95,99]. This suggests that increased expression of HERV-K (HML-2) in the neurons of ALS patients may contribute to the neurodegenerative process. In multiple sclerosis (MS), increased HERV-K (HML-2) expression is thought to be upregulated in the central nervous system [159,160]. This contributes to inflammation and immune activation [3]. In rheumatoid arthritis (RA), the mRNA of HERV-K (HML-2) *gag* gene was found to be significantly upregulated in RA patients, and this phenomenon was also confirmed in pemphigus vulgaris patients [124,161,162]. HERV-K (HML-2) has been associated with autoimmune diseases in several studies and may be utilized for therapeutic monitoring [62,116,163]. Several studies have also investigated the expression of HERV-K (HML-2) in psychiatric disorders [3]. It has been reported that brain tissue from patients with schizophrenia and bipolar disorder has higher expression of HERV-K (HML-2) than normal tissue and that the expression of HERV-K (HML-2) *gag* is relatively low in the blood of children with certain language disorders [3,144,164]. Many reports have studied the HERV-K (HML-2) expression, but little is known about its site-specific transcription. This is an important clue to identify direct interactions with disease. For this purpose, it is important to identify the location and expression of individual HERV-K (HML-2) that is not localized in the human reference genome. Several studies have applied next-generation sequencing technologies to determine the expression level and location of HERV-K (HML-2), identifying predominantly expressed loci that can be used to understand, detect, or inhibit disease. From the many studies reported to date, it seems clear that the HERV-K (HML-2) transcript is involved in human disease. However, the complex cellular interactions of host cells and the pathogenesis of these transcripts are still unclear, and further evidence needs to be accumulated.

## 10. Conclusions

The HERV-K (HML-2) subgroup is the most recent addition to the human genome and has played an important role in the evolution and diversity of the human genome. It has attracted the attention of many researchers due to its specialized structural and functional roles. To date, it remains polymorphic in human populations, and some have intact proviral sequences, so its association with human diseases is still being studied. Although various studies have shown its clear roles in the evolution and change of the human genome, its complex functional associations with disease remain to be elucidated. Due to its characteristics, it is still reported to cause genomic instability and contribute to various diseases and genetic variants. Tracking an individual’s HERV-K (HML-2) plays an important role in determining inter-individual susceptibility to certain diseases and identifying disease etiology. These relatively long repeat sequences are difficult to track, requiring the development of various NGS applications and bioinformatics tools. Furthermore, the complex regulatory network that controls its activity is not fully understood, and its correlation with the cell signaling system of the host cell needs to be further studied. We propose that revealing the enormous influence of HERV-K (HML-2) on the human genome will be an important key to understanding human disease and the role of other retrotransposons.

## Figures and Tables

**Table 1:** Typical HERVs classification.

	Class	Genus	Family	Subgroups
HERVs	Class I	Gammaretrovirus-like and epsilonretrovirus-like	HERV-W, HERV-H, HERV-F, HERV-P, HERV-E, HERV-R, HERV-T, HERV-I, ERV-FRD, ERV-FTD	
	Class II	Betaretrovirus-like	HERV-K	HML 1-11
	Class III	Spumaretrovirus-like	HERV-L	

## Data Availability

Not applicable.
